# Microbiological Diagnosis of Osteoarticular Infections: Comparison between BacT/Alert Bottles and Schaedler Broth

**DOI:** 10.1128/spectrum.00498-22

**Published:** 2022-04-12

**Authors:** Esther Gydé, Béate Heym, Florence Gillaizeau, Simon Marmor, Philippe Boisrenoult, Marlène Amara

**Affiliations:** a Centre Hospitalier de Versailles, Service de Biologie, Unité de Microbiologie, Le Chesnay, France; b Centre Hospitalier François Quesnay, Service de Biologie, Mantes La Jolie, France; c Groupe Hospitalier Diaconesses Croix Saint-Simon, Laboratoire des Centres de Santé et d’Hôpitaux d’Ile de France, Unité de Microbiologie, Paris, France; d Biofortis, Saint-Herblain, France; e Groupe Hospitalier Diaconesses Croix Saint-Simon, Service d’Orthopédie, Paris, France; f Centre Hospitalier de Versailles, Service d’Orthopédie, Unité Dominique Larrey, Le Chesnay, France; Johns Hopkins Hospital

**Keywords:** osteoarticular infection, BacT/Alert bottles, Schaedler broth, OAI

## Abstract

Microbiological diagnosis of osteoarticular infections (OAIs) is based on culture on several media. Experts recommend the use of liquid media, such as Schaedler broth, but many laboratories use blood culture media with automated detection instead for convenience. We aimed to evaluate the performance of culturing in BacT/Alert (bioMérieux) bottles for the microbiological diagnosis of OAI *versus* culturing in Schaedler broth. This prospective study was conducted on all osteoarticular specimens sent to the microbiology laboratories of the Versailles and Diaconesses Croix Saint-Simon hospitals between October 2016 and February 2017. Each sample was inoculated onto solid agar, into BacT/Alert bottles incubated for 14 days, and into a Schaedler broth incubated for 14 days with daily reading. The gold standard was defined as follow: OAI was diagnosed for a patient if at least two samples were positive for a nonskin microorganism and at least three for a cutaneous species. The times to detection were compared. A total of 1,616 specimens from 349 patients were collected. BacT/Alert bottles were significantly more sensitive than the Schaedler process for OAI diagnosis (114/135 OAI detected by BacT/Alert bottles; 91/135 OAI detected by Schaedler broth; +17.0% [95% confidence interval {CI}, 6.8%, 27.3%]; *P* = 0.0004). The time to detection was significantly shorter using BacT/Alert bottles (2.0 ± 2.2 days) than using Schaedler broth (4.6 ± 3.6 days, *P* < 0.0001). The culture of osteoarticular specimens in BacT/Alert bottles allows bacterial enrichment with an automated detection of positivity. Their use decreased detection time and increased sensitivity, making it a useful tool for the diagnosis of OAI that should be included among the recommended media.

**IMPORTANCE** Microbiological diagnosis of OAI is based on culture on several media. French experts recommend the use of liquid media such as Schaedler broth, but many laboratories use blood culture media with automated detection in substitution because it is more convenient. We report here a prospective multicentric study evaluating the performance of culture in BacT/Alert (bioMérieux) bottles for microbiological diagnosis of OAI in comparison with culture in Schaedler broth. A total of 1,616 osteoarticular specimens from 349 patients were collected and inoculated onto agar, into BacT/Alert aerobic and anaerobic bottles, and into a Schaedler broth. BacT/Alert bottles were significantly more sensitive than the Schaedler process for OAI diagnosis (+17.0% [95% CI, 6.8%, 27.3%], *P* = 0.0004). The time to detection was significantly shorter for the BacT/Alert bottles (2.0 ± 2.2 days) than for Schaedler broth (4.6 ± 3.6 days, *P* < 0.0001). This study suggests that the use of BacT/Alert bottles should be recommended in microbiological diagnosis of OAI.

## INTRODUCTION

Osteoarticular infections (OAIs) represent an important burden for patients and health care resources (1). The bacteria involved frequently belong to the skin flora, which complicates OAI diagnosis. Five samples of macroscopically different pathological anatomical sites are classically analyzed to distinguish the microorganisms responsible for infection from contaminating microorganisms (2–5). These samples are inoculated onto supplemented media (6, 7): aerobic and anaerobic Columbia blood agars incubated at 35°C, chocolate agar incubated at 35°C under a 5% CO_2_ atmosphere, and a liquid medium such as Schaedler broth (or brain heart infusion broth), which significantly improves the sensitivity of bacteriological culture (4, 8). Several laboratories have replaced these media with Bactec (Becton, Dickinson) or BacT/Alert (bioMérieux) aerobic and anaerobic bottles, which are more convenient. Indeed, these bottles are incubated for 14 days in an instrument that detects bacterial growth by CO_2_ emission. Positive bottles are microscopically examined and the cultures are seeded onto agars, whereas negative cultures require no additional time (9). Conversely, the use of Schaedler broth requires a daily visual check of broth turbidity.

We aimed to evaluate the performance of culturing in BacT/Alert bottles *versus* culturing in Schaedler broth for the microbiological diagnosis of OAI.

## RESULTS

After exclusion of 112 patients with fewer than three available samples, 349 patients (45 patients from one hospital and 304 from the other) were included: 52.4% were male (183/349), and the mean age was 67.1 years (standard deviation [SD] ±15.14). Most patients had osteosynthesis or prosthetic materials (83.4%; 291/349), and collected samples were mainly represented by hips (47.9%; 167/349) and knees (30.7%; 107/349) (Table 1). A total of 1,616 samples were examined, with a mean of 4.63 (±2.31) samples per patient. According to the gold standard, 135 out 349 patients (38.7%) were infected, mainly with Staphylococcus aureus (35.6% of OAI). A description of the microorganisms detected using solid agar, aerobic BacT/Alert bottles, anaerobic BacT/Alert bottles, combined BacT/Alert bottles, or Schaedler broth among the 1,616 samples is provided in Table 2.

**TABLE 1:** Demographic and pathological characteristics of the study patients

Characteristic	No. (%) of patients with indicated characteristic^*a*^		
	Infected patients (*n* = 135)	Noninfected patients (*n* = 214)	All patients (*n* = 349)
Sex			
Male	91 (67.4)	92 (43.0)	183 (52.4)
Female	44 (32.6)	122 (57.0)	166 (47.6)
			
Age (yrs) (mean ± SD)	66.8 (±15.83)	67.2 (±14.73)	67.1 (±15.14)
			
Osteosynthesis or prosthetic materials			
Yes	103 (76.3)	188 (87.9)	291 (83.4)
No	32 (23.7)	26 (12.1)	58 (16.6)
			
Joint			
Upper limb			
Shoulder	6 (4.4)	6 (2.8)	12 (3.4)
Humerus	1 (0.7)	0 (0.0)	1 (0.3)
Elbow	1 (0.7)	1 (0.5)	2 (0.6)
Ulna	1 (0.7)	0 (0.0)	1 (0.3)
Wrist	1 (0.7)	2 (0.9)	3 (0.9)
Finger	1 (0.7)	1 (0.5)	2 (0.6)
Lower limb			
Hip	54 (40.0)	113 (52.8)	167 (47.9)
Femur	2 (1.5)	4 (1.9)	6 (1.7)
Knee	32 (23.7)	75 (35.0)	107 (30.7)
Tibia	15 (11.1)	6 (2.8)	21 (6.0)
Ankle	2 (1.5)	5 (2.3)	7 (2.0)
Foot	15 (11.1)	0 (0.0)	15 (4.3)
Toe	2 (1.5)	0 (0.0)	2 (0.6)
Spine	2 (1.5)	1 (0.5)	3 (0.9)

a All values are number (%) of patients unless indicated otherwise.

**TABLE 2:** Description of microorganisms detected using solid agar, aerobic BacT/Alert bottles, anaerobic BacT/Alert bottles, combined BacT/Alert bottles, or Schaedler broth among the 1,616 samples

Microorganism detected	No. (%) of samples with detection				
	Positive solid agar	Positive aerobic BacT/ALert bottles	Positive anaerobic BacT/Alert bottles	Positive combined BacT/Alert bottles	Positive Schaedler broth
Staphylococcus species					
S. aureus	155 (9.6)	180 (11.1)	179 (11.1)	185 (11.4)	157 (9.7)
*S. capitis*	7 (0.4)	13 (0.8)	15 (0.9)	15 (0.9)	8 (0.5)
*S. caprae*	6 (0.4)	12 (0.7)	14 (0.9)	14 (0.9)	12 (0.7)
*S. cohnii*	0 (0.0)	3 (0.2)	1 (0.1)	3 (0.2)	0 (0.0)
S. epidermidis	45 (2.8)	87 (5.4)	85 (5.3)	96 (5.9)	74 (4.6)
S. hominis	1 (0.1)	3 (0.2)	0 (0.0)	3 (0.2)	0 (0.0)
* S. lugdunensis*	17 (1.1)	24 (1.5)	24 (1.5)	26 (1.6)	31 (1.9)
*S. pettenkoferi*	0 (0.0)	0 (0.0)	0 (0.0)	0 (0.0)	1 (0.1)
*S. saccharolyticus*	1 (0.1)	0 (0.0)	8 (0.5)	8 (0.5)	2 (0.1)
*S. warneri*	0 (0.0)	1 (0.1)	1 (0.1)	1 (0.1)	1 (0.1)
	
Streptococcus species					
S. agalactiae	11 (0.7)	18 (1.1)	18 (1.1)	19 (1.2)	16 (1.0)
*S. anginosus*	6 (0.4)	6 (0.4)	6 (0.4)	6 (0.4)	5 (0.3)
S. dysgalactiae	11 (0.7)	9 (0.6)	12 (0.7)	12 (0.7)	10 (0.6)
S. gallolyticus	7 (0.4)	8 (0.5)	8 (0.5)	8 (0.5)	5 (0.3)
S. mutans	1 (0.1)	4 (0.2)	4 (0.2)	4 (0.2)	0 (0.0)
S. oralis	7 (0.4)	11 (0.7)	11 (0.7)	11 (0.7)	10 (0.6)
S. pyogenes	13 (0.8)	13 (0.8)	13 (0.8)	13 (0.8)	6 (0.4)
	
*Enterococcus* species					
E. faecalis	24 (1.5)	30 (1.9)	31 (1.9)	32 (2.0)	32 (2.0)
E. faecium	3 (0.2)	3 (0.2)	4 (0.2)	4 (0.2)	3 (0.2)
	
Other Gram-positive cocci					
Dolosigranulum pigrum	2 (0.1)	5 (0.3)	3 (0.2)	5 (0.3)	3 (0.2)
Finegoldia magna	9 (0.6)	0 (0.0)	1 (0.1)	1 (0.1)	3 (0.2)
Granulicatella adiacens	4 (0.2)	0 (0.0)	0 (0.0)	0 (0.0)	0 (0.0)
Parvimonas micra	12 (0.7)	0 (0.0)	1 (0.1)	1 (0.1)	11 (0.7)
*Peptoniphilus harei*	0 (0.0)	0 (0.0)	5 (0.3)	5 (0.3)	0 (0.0)
	
Gram-positive bacilli					
Actinomyces europaeus	5 (0.3)	0 (0.0)	3 (0.2)	3 (0.2)	0 (0.0)
Actinomyces neuii	3 (0.2)	3 (0.2)	3 (0.2)	3 (0.2)	0 (0.0)
Actinobaculum schaalii	0 (0.0)	0 (0.0)	1 (0.1)	1 (0.1)	0 (0.0)
Corynebacterium amycolatum	0 (0.0)	0 (0.0)	0 (0.0)	0 (0.0)	1 (0.1)
Corynebacterium striatum	1 (0.1)	1 (0.1)	1 (0.1)	1 (0.1)	1 (0.1)
Corynebacterium tuberculostearicum	1 (0.1)	0 (0.0)	0 (0.0)	0 (0.0)	0 (0.0)
*Dermabacter hominis*	2 (0.1)	1 (0.1)	2 (0.1)	3 (0.2)	0 (0.0)
Lactobacillus rhamnosus	6 (0.4)	4 (0.2)	4 (0.2)	5 (0.3)	5 (0.3)
Cutibacterium acnes	37 (2.3)	5 (0.3)	40 (2.5)	40 (2.5)	29 (1.8)
Cutibacterium avidum	16 (1.0)	11 (0.7)	14 (0.9)	15 (0.9)	18 (1.1)
Trueperella bernardiae	3 (0.2)	0 (0.0)	1 (0.1)	1 (0.1)	0 (0.0)
	
Gram-negative bacteria					
Citrobacter koseri	11 (0.7)	11 (0.7)	10 (0.6)	11 (0.7)	10 (0.6)
Enterobacter cloacae	32 (2.0)	40 (2.5)	40 (2.5)	41 (2.5)	42 (2.6)
Escherichia coli	39 (2.4)	42 (2.6)	39 (2.4)	44 (2.7)	39 (2.4)
Klebsiella oxytoca	2 (0.1)	0 (0.0)	0 (0.0)	0 (0.0)	0 (0.0)
Klebsiella pneumoniae	8 (0.5)	11 (0.7)	12 (0.7)	12 (0.7)	12 (0.7)
Morganella morganii	6 (0.4)	5 (0.3)	8 (0.5)	8 (0.5)	7 (0.4)
Proteus mirabilis	11 (0.7)	12 (0.7)	10 (0.6)	12 (0.7)	13 (0.8)
Serratia marcescens	1 (0.1)	4 (0.2)	7 (0.4)	7 (0.4)	3 (0.2)
Pseudomonas aeruginosa	36 (2.2)	47 (2.9)	28 (1.7)	48 (3.0)	32 (2.0)
Acinetobacter spp.	0 (0.0)	4 (0.2)	0 (0.0)	4 (0.2)	0 (0.0)
Gardnerella vaginalis	11 (0.7)	0 (0.0)	0 (0.0)	0 (0.0)	2 (0.1)
Haemophilus parainfluenzae	3 (0.2)	0 (0.0)	0 (0.0)	0 (0.0)	0 (0.0)
Neisseria flava	2 (0.1)	2 (0.1)	2 (0.1)	2 (0.1)	2 (0.1)
	
Other anaerobes	11 (0.7)	3 (0.2)	8 (0.5)	8 (0.5)	3 (0.2)
	
Yeast spp.					
Candida albicans	0 (0.0)	2 (0.1)	0 (0.0)	2 (0.1)	0 (0.0)
Candida glabrata	0 (0.0)	1 (0.1)	3 (0.2)	3 (0.2)	0 (0.0)
Candida tropicalis	0 (0.0)	1 (0.1)	0 (0.0)	1 (0.1)	0 (0.0)
*Cyberlindnera rhodanensis*	1 (0.1)	1 (0.1)	0 (0.0)	1 (0.1)	7 (0.4)

The sensitivities of the BacT/Alert bottles and Schaedler broth for OAI diagnosis were evaluated according to the infecting bacteria (Table 3).

**TABLE 3:** Sensitivity of aerobic BacT/Alert bottles, anaerobic BacT/Alert bottles, combined BacT/Alert bottles, or Schaedler broth for OAI diagnosis

Microorganism responsible for OAI (no. of patients with OAI)	% Sensitivity (95% CI)			
	Aerobic BacT/Alert bottles	Anaerobic BacT/Alert bottles	Combined BacT/Alert bottles	Schaedler broth
Staphylococcus species				
S. aureus (48)	91.7 (80.0, 97.7)	91.7 (80.0, 97.7)	95.8 (85.7, 99.5)	85.4 (72.2, 93.9)
Coagulase-negative staphylococci (35)	71.4 (53.7, 85.4)	77.1 (59.9, 89.6)	85.7 (69.7, 95.2)	71.4 (53.7, 85.4)
*S. capitis* (2)	100.0 (15.8, 100.0)	100.0 (15.8, 100.0)	100.0 (15.8, 100.0)	100.0 (15.8, 100.0)
*S. caprae* (3)	66.7 (9.4, 99.2)	100.0 (29.2, 100.0)	100.0 (29.2, 100.0)	33.3 (0.8, 90.6)
S. epidermidis (20)	80.0 (56.3, 94.3)	80.0 (56.3, 94.3)	90.0 (68.3, 98.8)	80.0 (56.3, 94.3)
S. hominis (1)	100.0 (2.5, 100.0)	0.0 (0.0, 97.5)	100.0 (2.5, 100.0)	0.0 (0.0, 97.5)
*S. lugdunensis* (8)	62.5 (24.5, 91.5)	50.0 (15.7, 84.3)	62.5 (24.5, 91.5)	75.0 (34.9, 96.8)
*S. saccharolyticus* (2)	0.0 (0.0, 84.2)	100.0 (15.8, 100.0)	100.0 (15.8, 100.0)	0.0 (0.0, 84.2)
				
Streptococcus species (16)	87.5 (61.7, 98.5)	93.8 (69.8, 99.8)	93.8 (69.8, 99.8)	68.8 (41.3, 89.0)
S. agalactiae (4)	100.0 (39.8, 100.0)	100.0 (39.8, 100.0)	100.0 (39.8, 100.0)	100.0 (39.8, 100.0)
*S. anginosus* (1)	100.0 (2.5, 100.0)	100.0 (2.5, 100.0)	100.0 (2.5, 100.0)	100.0 (2.5, 100.0)
S. dysgalactiae (4)	50.0 (6.8, 93.2)	75.0 (19.4, 99.4)	75.0 (19.4, 99.4)	50.0 (6.8, 93.2)
S. gallolyticus (2)	100.0 (15.8, 100.0)	100.0 (15.8, 100.0)	100.0 (15.8, 100.0)	50.0 (1.3, 98.7)
S. mutans (1)	100.0 (2.5, 100.0)	100.0 (2.5, 100.0)	100.0 (2.5, 100.0)	0.0 (0.0, 97.5)
S. oralis (2)	100.0 (15.8, 100.0)	100.0 (15.8, 100.0)	100.0 (15.8, 100.0)	100.0 (15.8, 100.0)
S. pyogenes (3)	100.0 (29.2, 100.0)	100.0 (29.2, 100.0)	100.0 (29.2, 100.0)	66.7 (9.4, 99.2)
				
*Enterococcus* species (10)	80.0 (44.4, 97.5)	90.0 (55.5, 99.8)	90.0 (55.5, 99.8)	90.0 (55.5, 99.8)
E. faecalis (9)	77.8 (40.0, 97.2)	88.9 (51.8, 99.7)	88.9 (51.8, 99.7)	88.9 (51.8, 99.7)
E. faecium (1)	100.0 (2.5, 100.0)	100.0 (2.5, 100.0)	100.0 (2.5, 100.0)	100.0 (2.5, 100.0)
				
Other Gram-positive cocci				
*D. pigrum* (1)	100.0 (2.5, 100.0)	100.0 (2.5, 100.0)	100.0 (2.5, 100.0)	100.0 (2.5, 100.0)
*F. magna* (3)	0.0 (0.0, 70.8)	0.0 (0.0, 70.8)	0.0 (0.0, 70.8)	33.3 (0.8, 90.6)
*G. adiacens* (1)	0.0 (0.0, 97.5)	0.0 (0.0, 97.5)	0.0 (0.0, 97.5)	0.0 (0.0, 97.5)
P. micra (2)	0.0 (0.0, 84.2)	0.0 (0.0, 84.2)	0.0 (0.0, 84.2)	100.0 (15.8, 100.0)
*P. harei* (1)	0.0 (0.0, 97.5)	100.0 (2.5, 100.0)	100.0 (2.5, 100.0)	0.0 (0.0, 97.5)
				
Gram-positive bacilli				
*A. europaeus* (2)	0.0 (0.0, 84.2)	50.0 (1.3, 98.7)	50.0 (1.3, 98.7)	0.0 (0.0, 84.2)
*A. neuii* (1)	100.0 (2.5, 100.0)	100.0 (2.5, 100.0)	100.0 (2.5, 100.0)	0.0 (0.0, 97.5)
*D. hominis* (2)	0.0 (0.0, 84.2)	0.0 (0.0, 84.2)	50.0 (1.3, 98.7)	0.0 (0.0, 84.2)
L. rhamnosus (1)	100.0 (2.5, 100.0)	100.0 (2.5, 100.0)	100.0 (2.5, 100.0)	100.0 (2.5, 100.0)
*C. acnes* (9)	11.1 (0.3, 48.2)	77.8 (40.0, 97.2)	77.8 (40.0, 97.2)	55.6 (21.2, 86.3)
*C. avidum* (3)	100.0 (29.2, 100.0)	100.0 (29.2, 100.0)	100.0 (29.2, 100.0)	100.0 (29.2, 100.0)
*T. bernardiae* (1)	0.0 (0.0, 97.5)	0.0 (0.0, 97.5)	0.0 (0.0, 97.5)	0.0 (0.0, 97.5)
				
Gram-negative bacteria				
*Enterobacteriaceae* (29)	93.1 (77.2, 99,2)	96.6 (82.2, 99.9)	100.0 (88.1, 100.0)	93.1 (77.2, 99.2)
*C. koseri* (3)	100.0 (29.2, 100.0)	100.0 (29.2, 100.0)	100.0 (29.2, 100.0)	100.0 (29.2, 100.0)
E. cloacae (10)	100.0 (69.2, 100.0)	100.0 (69.2, 100.0)	100.0 (69.2, 100.0)	100.0 (69.2, 100.0)
E. coli (7)	85.7 (42.1, 99.6)	100.0 (59.0, 100.0)	100.0 (59.0, 100.0)	85.7 (42.1, 99.6)
K. oxytoca (1)	0.0 (0.0, 97.5)	0.0 (0.0, 97.5)	0.0 (0.0, 97.5)	0.0 (0.0, 97.5)
K. pneumoniae (3)	100.0 (29.2, 100.0)	100.0 (29.2, 100.0)	100.0 (29.2, 100.0)	100.0 (29.2, 100.0)
M. morganii (3)	66.7 (9.4, 99.2)	100.0 (29.2, 100.0)	100.0 (29.2, 100.0)	66.7 (9.4, 99.2)
P. mirabilis (4)	100.0 (39.8, 100.0)	75.0 (19.4, 99.4)	100.0 (39.8, 100.0)	100.0 (39.8, 100.0)
S. marcescens (2)	50.0 (1.3, 98.7)	100.0 (15.8, 100.0)	100.0 (15.8, 100.0)	50.0 (1.3, 98.7)
				
Nonfermenting bacilli (17)	88.2 (63.6, 98.5)	47.1 (23.0, 72.2)	88.2 (63.6, 98.5)	58.8 (32.9, 81.6)
P. aeruginosa (16)	87.5 (61.7, 98.5)	50.0 (24.7, 75.3)	87.5 (61.7, 98.5)	62.5 (35.4, 84.8)
Acinetobacter spp. (1)	100.0 (2.5, 100.0)	0.0 (0.0, 97.5)	100.0 (2.5, 100.0)	0.0 (0.0, 97.5)
				
Other Gram-negative bacteria				
*G. vaginalis* (2)	0.0 (0.0, 84.2)	0.0 (0.0, 84.2)	0.0 (0.0, 84.2)	50.0 (1.3, 98.7)
*H. parainfluenzae* (1)	0.0 (0.0, 97.5)	0.0 (0.0, 97.5)	0.0 (0.0, 97.5)	0.0 (0.0, 97.5)
*N. flava* (1)	100.0 (2.5, 100.0)	100.0 (2.5, 100.0)	100.0 (2.5, 100.0)	100.0 (2.5, 100.0)
Gram-negative anaerobes (4)	25.0 (0.6, 80.6)	75.0 (19.4, 99.4)	75.0 (19.4, 99.4)	25.0 (0.6, 80.6)
Yeasts (3)	33.3 (0.8, 90.6)	33.3 (0.8, 90.6)	66.7 (9.4, 99.2)	33.3 (0.8, 90.6)
C. albicans (1)	100.0 (2.5, 100.0)	0.0 (0.0, 97.5)	100.0 (2.5, 100.0)	0.0 (0.0, 97.5)
C. glabrata (1)	0.0 (0.0, 97.5)	100.0 (2.5, 100.0)	100.0 (2.5, 100.0)	0.0 (0.0, 97.5)
*C. rhodanensis* (1)	0.0 (0.0, 97.5)	0.0 (0.0, 97.5)	0.0 (0.0, 97.5)	100.0 (2.5, 100.0)

The sensitivities of the two media for OAI diagnosis were compared for the most frequently occurring microorganisms (microorganisms responsible for more than five OAIs). There was no statistically significant difference for Staphylococcus epidermidis OAI (+10.0% [95% confidence interval {CI}, −13.6%, 33.6%], *P* = 0.41), Cutibacterium acnes OAI (+22.2% [−18.8%, 63.3%], *P* = 0.29), and Staphylococcus lugdunensis OAI (−12.5% [−54.0%, 29.0%], *P* = 0.56). Although statistical significance was not reached, there was a tendency for higher sensitivity of the BacT/Alert bottles for S. aureus OAI (+10.4% [CI, −1.5%, 22.3%], *P* = 0.086) and Pseudomonas aeruginosa OAI (+25.0% [−2.4%, 52.4%], *P* = 0.074). Considering microorganisms by categories, there was a higher sensitivity of BacT/Alert bottles for nonfermenting bacillus OAI (+29.4% [CI, 2.3%, 56.5%], *P* = 0.034) and streptococcal species OAI (+25.0% [3.8%, 46.2%], *P* = 0.021). There was no statistically significant difference for coagulase-negative staphylococcus OAI (+14.3% [CI, −5.3%, 33.9%], *P* = 0.15).

Overall (for all microorganisms), the sensitivities for OAI diagnosis were 84.4% (CI, 78.3%, 90.6%) using the combined BacT/Alert bottles (114/135 OAI detected) and 67.4% (59.5%, 75.3%) using Schaedler broth (91/135 OAI detected), resulting in a difference of +17.0% with a 95% adjusted CI of 6.8%, 27.3% (*P = *0.0004). The lower limit of the 95% CI was greater than the predefined noninferiority margin and even greater than zero. Thus, the use of combined BacT/Alert bottles was not inferior to the Schaedler process for OAI diagnosis and beyond that demonstrated a significantly higher sensitivity than the Schaedler process for OAI diagnosis.

The time to detection was significantly shorter when BacT/Alert bottles were used (2.0 ± 2.2 days) than when Schaedler broth was used (4.6 ± 3.6 days, *P* < 0.0001).

## DISCUSSION

S. aureus was the microorganism most frequently responsible for OAI in our study (35.6%), concordant with literature (1, 10). There was no significant difference in sensitivity between BacT/Alert bottles and Schaedler broth for OAI related to the most frequently occurring microorganisms, although there was a tendency toward better sensitivity of the BacT/Alert bottles for S. aureus OAI (*P* = 0.086) and P. aeruginosa OAI (*P* = 0.074).

Our assessment of the noninferiority of the sensitivity shows that the sensitivity of the BacT/Alert bottles for OAI diagnosis is not less than but even higher than that of Schaedler broth, suggesting that the BacT/Alert bottles can be used instead of broth for OAI diagnosis. The sensitivity of both methods for diagnosis of OAI related to specific microorganisms such as yeasts, Gram-positive bacilli, or anaerobic bacteria was very weak, highlighting the importance of the use of solid agars, which is the only way to estimate bacterial load. In addition, liquid media, such as Schaedler broth and BacT/Alert bottles, can induce competition between microorganisms and conceal certain bacteria that exhibit poor growth in polymicrobial samples (11).

### Limitations of the study.

We had only very few positive samples for each microorganism and thus could not compare the sensitivities of the two methods species by species, due to low statistical power. We focused on microorganisms responsible for more than five OAIs, and this is a limitation to our study, especially for Enterococcus faecalis, *C. acnes*, and *S. lugdunensis*, which are responsible for fewer than 10 OAIs. Studies with a larger number of samples targeting specific microorganisms responsible for OAI could be informative.

### Conclusions.

Although several studies have demonstrated the benefits of blood culture bottles (8, 9, 12–15), this study is the first to include a large number of samples. Our results show that this automated method, less restrictive than the daily reading of broth, is faster for the detection of pathogens and more sensitive. Although BacT/Alert bottles are more expensive than Schaedler broth, the reduced turnaround time in the laboratory leads to a reduced length of stay for patients in the orthopedic unit, which is cost-effective for the hospital. Finally, the use of an automated method could lead to better and earlier diagnosis, hence improving patient care and adding medical value.

## MATERIALS AND METHODS

This prospective study was conducted over a 5-month period (October 2016 to February 2017) and focused on all osteoarticular specimens sent to the microbiology laboratories of two French hospitals belonging to the Reference Center for Complex Osteoarticular Infections (CRIOAC) of the Ile-de-France region. An exclusion criterion was applied: patients with fewer than three available samples were excluded from the study, since it did not fit in our gold standard definition (see description below). Cultures were incubated for 5 to 14 days in accordance with French microbiology recommendations (6).

After the addition of 5 mL of sterile water and grinding (IKA Ultra Turrax grinder, power 9 for 5 min, except for synovial fluid), each sample was inoculated onto agar (Columbia blood agar incubated for 5 days in an aerobic atmosphere or for 10 days in an anaerobic atmosphere at 35°C, chocolate agar incubated for 5 days in a 5% CO_2_ atmosphere at 35°C), into aerobic and anaerobic BacT/Alert bottles (FA and FN) incubated for 14 days in the BacT/Alert 3D (bioMérieux), and into Schaedler broth (bioMérieux) incubated for 14 days at 35°C with daily visual reading. Each positive BacT/Alert bottle culture and both positive and negative Schaedler broth cultures at day 14 were inoculated onto agar. Positive subcultures were identified by mass spectrometry (MALDI Biotyper; Bruker Daltonics, Bremen, Germany), and the time to microorganism detection was recorded.

### Statistical analysis.

Samples were considered to be positive for a microorganism if at least one microorganism was found in at least one of the culture media. The gold standard was defined according to French microbiology recommendations (6) and British orthopedic guidelines (2): OAI was diagnosed for a patient if at least two samples were positive for a nonskin microorganism and at least three for a cutaneous species.

The sensitivity of each medium for OAI diagnosis was examined. Noninferiority of the BacT/Alert method was assessed relative to the Schaedler method with a predetermined lower limit of noninferiority margin of −5% (mixed logistic model, Dunnett adjustment). The term “combined BacT/Alert bottles” was used when we considered the combined use of aerobic and anaerobic bottles.

Pairwise comparisons of the times to microorganism detection were performed for 109 positive samples (Wilcoxon signed rank test, Bonferroni-Holm adjustment). When one of the methods could not detect a microorganism, a value of infinity was attributed; when none of the methods could detect a microorganism, a value of zero was attributed.

Statistical analyses were performed using SAS software version 9.4 (SAS Institute, Inc., Cary, NC, USA).

### Ethics.

According to French law at the time of the start of the study and in accordance with the ethical standards of our hospitals’ institutional review boards (Committee for the Protection of Human Subjects), informed consent and ethics approval were not yet required for this observational study, which did not modify existing diagnostic or therapeutic strategies.
